# Astatine Facing Janus: Halogen Bonding vs. Charge-Shift Bonding

**DOI:** 10.3390/molecules26154568

**Published:** 2021-07-28

**Authors:** Serigne Sarr, Julien Pilmé, Gilles Montavon, Jean-Yves Le Questel, Nicolas Galland

**Affiliations:** 1 Laboratoire CEISAM, UMR CNRS 6230, Université de Nantes, 2 Rue de la Houssinière, 44322 Nantes, France; Serigne.Sarr1@etu.univ-nantes.fr (S.S.); jean-yves.le-questel@univ-nantes.fr (J.-Y.L.Q.); 2 Laboratoire de Chimie Théorique, UMR CNRS 7616, Sorbonne Université, 4 Place Jussieu, 75005 Paris, France; julien.pilme@sorbonne-universite.fr; 3 Laboratoire SUBATECH, UMR CNRS 6457, IN2P3/EMN Nantes/Université de Nantes, 4 Rue Alfred Kastler, 44307 Nantes, France; montavon@subatech.in2p3.fr

**Keywords:** astatine, halogen-bond interactions, spin–orbit coupling, charge-shift bonds, QTAIM, ELF, local electrophilicity

## Abstract

The nature of halogen-bond interactions was scrutinized from the perspective of astatine, potentially the strongest halogen-bond donor atom. In addition to its remarkable electronic properties (e.g., its higher aromaticity compared to benzene), C_6_At_6_ can be involved as a halogen-bond donor and acceptor. Two-component relativistic calculations and quantum chemical topology analyses were performed on C_6_At_6_ and its complexes as well as on their iodinated analogues for comparative purposes. The relativistic spin–orbit interaction was used as a tool to disclose the bonding patterns and the mechanisms that contribute to halogen-bond interactions. Despite the stronger polarizability of astatine, halogen bonds formed by C_6_At_6_ can be comparable or weaker than those of C_6_I_6_. This unexpected finding comes from the charge-shift bonding character of the C–At bonds. Because charge-shift bonding is connected to the Pauli repulsion between the bonding σ electrons and the σ lone-pair of astatine, it weakens the astatine electrophilicity at its σ-hole (reducing the charge transfer contribution to halogen bonding). These two antinomic characters, charge-shift bonding and halogen bonding, can result in weaker At-mediated interactions than their iodinated counterparts.

## 1. Introduction

Many standard classifications in chemistry cross the group of p-elements, especially the chemical families known as pnictogens, chalcogens, and halogens. The pnictogen, chalcogen, and halogen elements are commonly involved in non-covalent interactions, sometimes gathered under the name of σ-hole interactions [1,2]. Bound to an electron-withdrawing group, these elements display a region of electron deficiency, the so-called σ-hole, which can induce highly directional attractive interactions with a nucleophilic region from another, or from the same, molecular entity. These interactions are better known, since the 2000s, as pnictogen, chalcogen, and halogen bonds, the latter being increasingly used in materials science, supramolecular chemistry, organocatalysis, chemical biology, and medicinal chemistry [3,4,5,6,7,8,9,10]. Among this framework, halobenzenes are an interesting class of halogen-bond donors, R–X, because halogen atoms X can be easily introduced into a benzene ring at different positions. In addition, these compounds provide a convenient platform for introducing substituents and investigating, experimentally and theoretically, their effects on the ability of X to mediate halogen-bond interactions (i.e., halogen-bond tuning) [11,12,13,14,15,16,17]. Currently, it is commonly recognized that the interaction strength increases as R is more electron-withdrawing, and for a given R, in the order X = F < Cl < Br < I, following the trend in the halogens’ polarizabilities (and decreasing electronegativities) [2,10,18].

Among the halogens, astatine has long been expected to be the strongest halogen-bond donor atom [19,20], but this assumption is particularly challenging to prove experimentally, since astatine is the rarest element naturally occurring on Earth. Beyond the very small amounts available, which are produced in particle accelerators, the lack of suitable spectroscopic tools severely limits the investigations of astatine’s chemical and physical properties [21]. Very recently, several of the current authors reported the first experimental characterization of halogen bonds (XBs) mediated by astatine [22]. That work and its following support a highest donating ability of astatine compared to iodine [23]. The adducts between astatine monoiodide (AtI) and ten Lewis bases were identified through the comparison of measured formation constants and those calculated using relativistic quantum mechanical methods. Indeed, astatine (Z = 85) is a heavy element and, as such, it is subject to significant relativistic effects. The scalar-relativistic effects are essentially associated with the relativistic mass increase of the electrons, resulting from their high speed near the nucleus. The main spin-dependent effect is the spin–orbit coupling (SOC), i.e., the interaction of the electron spin with magnetic fields generated by other charged particles in relative motion, leading to the coupling between electron spin and orbital momentum. The SOC effects can be rather important on the characteristics of XBs formed by astatine. In a theoretical study focused on XAt^…^NH_3_ complexes (X = F–At), the SOC was found to weaken the interaction by more than 20% when X = F or At [24]. At-mediated XBs can be enhanced as well with the inclusion of the spin–orbit interaction in the relativistic calculations [25].

Using state-of-the-art relativistic quantum mechanical methods, Alvarez-Thon and co-workers have recently scrutinized how relativity affects the electronic structure of hexahalogened benzenes, C_6_X_6_ (X = F–At) [26]. As expected, the SOC had no or trifling effect on the aromaticity in C_6_F_6_, C_6_Cl_6_, and C_6_Br_6_. Conversely, the influence became noticeable in C_6_I_6_ and had a dramatic effect in C_6_At_6_. Indeed, the significant contribution of the spin–orbit interaction to the current electronic flow around the molecular framework makes the hypothetical C_6_At_6_ molecule more aromatic than the archetypical aromatic benzene, according to the calculated ring current strengths. In this work we propose to study how significantly the SOC effect can impact on the ability of C_6_At_6_ to form XBs. More precisely, the use of quantum chemical topology (QCT) notably helps to probe the nature of C–At bonds. For comparison, the study of the effects of SOC on the iodinated analogue, C_6_I_6_, was also made. The disclosed trends were then tested through the study of another aromatic compound, in connection with the applications currently envisaged for the 211 radioisotope of astatine in nuclear medicine [21].

## 2. Materials and Methods

The two-component relativistic density functional theory (DFT), which was proved to be accurate for investigating At-containing systems [27,28], requires the replacement of the orbital representation by spinors that are complex vector functions of two components (2c). The Generalized Kohn–Sham (GKS) method, implemented in the Gaussian 16 rev. A.03 program [29], takes advantage of the relativistic pseudo-potentials containing scalar and spin-dependent terms to treat the electron correlation and the relativistic effects on an equal footing. In a recent benchmark study focusing on At-compounds [30], the PW6B95 hybrid functional clearly emerged as one of the best performing ones among 36 tested DFT functionals. Moreover, PW6B95 has also been validated as reliable for investigating compounds stabilized by At-mediated halogen bonds [22,23,25], notably according to results obtained with the gold-standard CCSD(T) method [24,31,32]. In the benchmark study, it was also noticed, regarding the complexes stabilized by At-mediated interactions, that results may be slightly improved by the inclusion of a dispersion correction [30]. The D3 version of Grimme’s dispersion correction, using the Becke–Johnson damping function GD3BJ, was selected [33]. The small core pseudo-potential ECP60MDF was used for the At atoms, and the ECP28MDF one for the I atoms [34,35]. The explicitly treated electrons were described by a set of triple zeta quality basis sets, abbreviated as TZVPPD. It includes the dhf-TZVPPD-2c basis sets for the At and I atoms [36], and the def2-TZVPP basis sets for the remaining atoms [37], with diffuse functions being added for non-H atoms [38]. To evaluate the SOC effects (∆SO) on the properties of the studied species, geometry optimizations and frequency calculations were performed at both the 2c-relativistic DFT level and the scalar-relativistic DFT level (the one-electron spin–orbit terms were removed from the pseudo-potentials). Vibrational harmonic frequencies were used to establish the nature of the structures (minima vs. transition states). The energies of the XB complexes were corrected from the basis set superposition error (BSSE) using the counterpoise (CP) method [39], and the corresponding interaction energies were calculated using the super-molecule approach. In order to assess the reliability of previously calculated geometries and interaction energies, CP-corrected MP2/TZVPPD geometry optimizations were performed at the scalar-relativistic (sr) level. Further sr-MP2/TZVPPD and 2c-MP2/TZVPPD single-point calculations, using the resolution of the identity technique in the TURBOMOLE program package [40], were respectively performed on sr- and 2c-PW6B95/TZVPPD geometries. Note that the core electrons were frozen for all MP2 calculations (including the semi-core 4*s*4*p*4*d* electrons of I and 5*s*5*p*5*d* electrons of At). For calculating the local electrophilicity of the studied species, we used values obtained by the finite difference method [41]. The Fukui function for localizing electrophilic sites, *f* ^+^ (*r*), is obtained as:

*f* ^+^ (*r*) ≈ *ρ*_N+1_(*r*) − *ρ*_N_(*r*)

where *ρ*_N_ and *ρ*_N+1_ are the electron densities of the neutral species (*N* electrons) and of the corresponding anion (*N* + 1 electrons) at the geometry of the neutral species. Furthermore, the electronegativity, *χ* = ½(IE + EA), and the hardness, *η* = (IE − EA), were obtained from the vertical ionization energy (IE) and electron affinity (EA) values.

Introduced by Becke and Edgecombe [42], the electron localization function (ELF) is a signature of the distribution of electronic pairs. The analysis of its topology is a powerful tool for the characterization of bonding schemes [43]. The physical space is divided into electronic volumes or basins, which are localized around the maxima of the function and are separated by zero flux surfaces. The ELF partition assigns the electron density between core basins around nuclei A, C(A), and valence basins. The latter are divided into non-bonding basins, V(A), usually corresponding to lone-pairs, and bonding basins, V(A_1_, A_2_), characterizing the covalent character of the bond between two atoms, A_1_ and A_2_. The spatial distribution of the valence basins closely matches the non-bonding and bonding domains of the valence shell electron pair repulsion (VSEPR) theory [44]. The integration of the electron density over the volume of the basins provides the average basin populations, which can be understood as arising from Lewis resonant structures [45]. The statistical analysis of basin populations through the definitions of the variance and the covariance matrices, provides information about the electron delocalization between basins. Furthermore, it was found from experimental and theoretical works that the quantum theory of atoms in molecules (QTAIM), formulated by Bader, provides a route to analyze, evaluate, and classify the nature of chemical bonds and interactions [46,47,48]. In the QTAIM theory, the function of interest is the electron density *ρ*(*r*). Each atom within a molecule corresponds to a basin, and the integration of the electron density over the basin volume provides an atomic population. The critical points of the electron density and their connectivity provide a characterization of the topology. The stationary point of the electron density along the line of the minimum electron density connecting two atoms, named the bond critical point (BCP), plays a central role in the QTAIM classification [49]. For example, the electron density at the BCP, *ρ*_b_, is in general large in the case of “shared-shell” interactions (mainly covalent bonds) and generally lower than 0.10 a.u. in the case of “closed-shell” interactions (e.g., ionic bonds and weak interactions) [47,50]. Furthermore, a negative value of the Laplacian of the density, ∇^2^*ρ*_b_, indicates a local concentration of charge at the BCP, which is typical of shared-shell interactions. Conversely, a positive value of ∇^2^*ρ*_b_ indicates a depletion of charge that characterizes closed-shell interactions [49]. Another widely used descriptor is the delocalization index (DI), noted δ(A, B). It measures the electron pair covalently sharing between two topological atoms A and B [51]. Details on the extension of the ELF and QTAIM topological analyses in the framework of 2c-relativistic DFT calculations can be found in [32,52,53]. All the topological analyses were carried out using a modified version of the TopChem2 program [54].

## 3. Results and Discussion

As indicated previously, we first sought to reveal the relativistic effects on the bonding patterns in the C_6_At_6_ species and also in C_6_I_6_ for comparative purposes. We will focus more specifically on the influence of the spin–orbit interaction, at the origin of the significant effect shown by Alvarez-Thon and co-workers (i.e., perastatobenzene becoming more aromatic than benzene) [26]. The tools of QCT, and especially the QTAIM and ELF topological analyses, have previously demonstrated their benefits for such studies [55,56,57,58,59].

### 3.1. Topological Analyses of the C_6_X_6_ XB Donors

**Figure 1:**
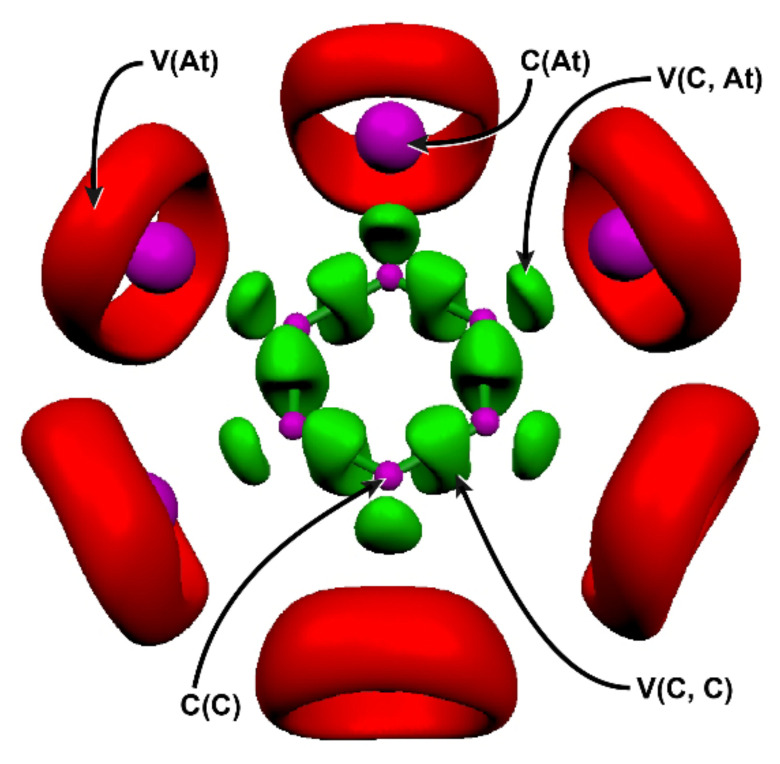
ELF localization domains for the C_6_At_6_ species (ELF isosurface = 0.75) at the 2c-PW6B95-D3/TZVPPD level of theory. Color code: magenta for core basins, red for valence non-bonding basins, and green for bonding basins.

Figure 1 shows the ELF localization domains determined for C_6_At_6_, which is also representative of the topology obtained for C_6_I_6_. Each atom has one core basin, C(C) for the carbon atoms and C(X) for the halogen atoms. One bonding basin is found between each pair of adjacent carbon atoms, V(C, C), as well as between the bounded carbon and halogen atoms, V(C, X). Finally, each halogen atom displays one non-bonding valence basin, V(X), describing all its lone-pairs. The electron populations of most ELF basins and some other descriptors calculated at the 2c-PW6B95-D3/TZVPPD level of theory for C_6_At_6_ and C_6_I_6_ are gathered in Table 1.

The populations for C(X) + V(X), ~24.3 e, were very close to the sum of 18 core electrons (i.e., the outermost core electrons that were not included in the pseudo-potential) and 6 electrons associated to three lone-pairs for each halogen. The V(C, X) bonding basins showed populations, ~1.7 e, below the typical value of two electrons expected for a pure single covalent bond. This depleted electron population indirectly points to an additional component to covalent bonding. However, for C–At bonds, it cannot be attributed to an ionic character as we will see hereafter. As expected, the populations of the V(C, C) basins, ~2.9 e, were intermediate between those of a single and a double bond (2 vs. 4 electrons). For instance, the bonding electron population for benzene C–C bonds is known from previous ELF analyses to be ~2.8 e [60,61]. In the framework of ELF topological analyses, a measure of electron delocalization is provided by the electron population N of the bonding basins, but also by its variance σ^2^. The variance for an ELF basin represents the electron fluctuation between the basin and the rest of the molecule, and it is useful to define the relative fluctuation λ = σ^2^/N. In the benzene molecule, characterized by a highly delocalized π system, the relative fluctuation of the V(C, C) basins is about 46% [60,61]. Similar values (48%) were obtained for the V(C, C) basins in C_6_At_6_ and C_6_I_6_ species.

The most striking result is the much higher value of λ for the V(C, X) basins and, in particular, for the C–At bond: 63% of the 1.70 bonding electrons fluctuated between each V(C, At) basin and other regions of the molecule. For a σ bond, a large variance in addition to a depleted basin population is indicative of a significant charge-shift (CS) bonding character. CS bonds have been proposed as a third bonding modality, alongside the two traditional covalent and ionic bond families [62,63]. Considering a bond of the general type R–X, the bonding itself owes its stability from large and dynamic fluctuations of the bonding electron-pair, which can be pictured through resonance between the covalent and ionic Lewis structures: R| X^+^ ↔ R–X ↔ ^+^R |X^−^. The CS mechanism originates in Pauli repulsions between the bonding electrons and the lone-pairs adjacent to the bond, which have the same symmetry as the bond (here, the halogen σ lone-pair). This effect weakens the covalent contribution to the bonding and is referred to as the lone pair bond-weakening effect (LPBWE). The bond stabilization is achieved by an optimal covalent-ionic mixing, resulting in a tremendous resonance energy. The elements that are prone to CS bonding are compact electronegative and/or lone-pair-rich atoms, albeit with moderate electronegativities [64]. Astatine has notably a demonstrated potential to form bonds of CS type [25,52,59], notably with carbon atoms [32,52].

More decisive elements are provided by QTAIM analyses. Some descriptors of the nature of C–X bonds are presented in Table 2, while those related to C–C bonds are gathered in Table S1 in Supplementary Materials. It is worth noting that, at the BCP of the C–C bonds in C_6_At_6_ and C_6_I_6_ species, the electron density was larger than 0.2 a.u. and the Laplacian of the electron density was negative. According to the standard QTAIM classification [47,50], these are two characteristics of shared-shell interactions, i.e., mainly covalent bonds. Furthermore, the delocalization index, which is considered as a measure of the sharing of electron-pairs between atoms [50,65], is very informative regarding the nature of C–C bonds. The value of δ(C, C), ~1.36, was thereby intermediate between the formal bond orders of a single and a double bond, supporting the picture of aromatic rings built of conjugated covalent C–C bonds. The situation regarding the C–I bonds also seemed rather clear, according to the results of Table 2. $\nabla^{2}\rho_{b}$ was negative and, although the electron density was somewhat weak at the BCP ($\rho_{b}$ = 0.13 a.u.), the δ(C, I) value of 1.11 also supported their designation as covalent single bonds. Conversely, the QTAIM descriptors for the C–At bonds may appear confusing. At the BCP and according to the standard QTAIM classification, the positive value of $\nabla^{2}\rho_{b}$ and the value of 0.10 a.u. of $\rho_{b}$ would indicate closed-shell interactions between C and At atoms, i.e., bonds were dominated by an ionic character. However, this view is contradicted by the computed atomic charge for astatine, which was not so large, 0.19 e, and identical to that of iodine in the covalent C–I bonds of C_6_I_6_. Furthermore, the delocalization index is found in the literature to have a value approaching to zero for ionic bonds, meanwhile it shows values approaching the formal bond order for covalent and CS bonds [65,66]. The value of δ(C, At), 0.99, disagrees with the picture of a mainly ionic bond. In fact, an important feature was disclosed: the nature of these C–At bonds did not fit with the standard QTAIM classification. This is a characteristic of CS bonds [63,67].

The ability of chemical elements to form CS bonds originates in part from the compactness of their valence orbitals [62]. The SOC enhances the astatine propensity for CS bonding [52,59], most probably due to the dominant shrinkage of the 6p_1/2_ shell upon inclusion of SOC, by ~0.2 Å with respect to the 6p_z_ orbital [68]. It is therefore of interest to scrutinize the SOC effects on the topological descriptors in order to assess the CS character of C–At bonds in C_6_At_6_. In Table 2, the SOC effect (∆SO) is defined as the difference between the results of 2c-PW6B95-D3/TZVPPD and sr-PW6B95-D3/TZVPPD calculations. The CS mechanism is associated to a weakening of the covalent contribution to the bonding. In the QTAIM approach, the ratio between the potential (V) and positive definite kinetic (G) energy densities at the BCP, |V_b_|/G_b_, reflects the magnitude of covalency [50,69]. This ratio decreased upon SOC by ~4%, and the electron density, too, by ~11% at the BCP (Table 2). The synchronized increase in $\nabla^{2}\rho_{b}$ widened the gap with the QTAIM category of covalent bonds (i.e., shared-shell interaction). Further clues are provided by looking at the SOC effects on the ELF descriptors (Table 1). Associated to a slight decrease in the electron population of V(C, At), an increase by ~1% of the relative fluctuation for the bonding basin was obtained, in line with an increased covalent-ionic resonance. All these changes, although quantitatively moderate, support a noticeable CS character for C–At bonds in C_6_At_6_, enhanced with the spin–orbit interaction.

The Pauli repulsion operating between bonding and non-bonding σ electrons is a physical origin of CS bonding [62,63]. Because its lone-pairs were involved, consequences are expected on the halogen atom’s ability to act as a Lewis base or as a Lewis acid in the case of XB interactions. The conceptual density functional theory provides appealing and general-purpose models for chemical reactivity. Several of the current authors recently proposed to use the local electrophilicity *ω*^+^(*r*) to characterize σ-hole interactions, and more precisely *ω*^+^_S,max_, which is the maximum value at the σ-hole on the molecular surface [31]. *ω*^+^_S,max_ is intended to probe the charge transfer contribution to the formation of XB interactions. *ω*^+^(*r*) is defined as the product between the global electrophilicity index *ω* (*χ*^2^/2*η*) and the *f*^+^(*r*) Fukui function, which shows the distribution of an infinitesimal charge added to the system [41]. A high value of *ω*^+^(*r*) indicates therefore an electrophilic zone. Figure S1 in the Supplementary Materials displays *ω*^+^(*r*) at the molecular surface of C_6_At_6_ (and C_6_I_6_), highlighting the structure of the σ-holes at the astatine (and iodine) sites. It is worth noting that *ω*^+^_S,max_ was demonstrated to be a better descriptor for anticipating the SOC effects on XBs mediated by astatine than the well-known *V*_S,max_ descriptor, i.e., the maximum value of the molecular electrostatic potential calculated at the σ-hole [24,25]. Associated with the strengthening upon SOC of the CS character of C–At bonds in C_6_At_6_, the local electrophilicity at the astatine σ-hole significantly decreased, by ~20% according to the *ω*^+^_S,max_ descriptor (Figure S1). Next, we studied how the SOC effect on the nature of C–At bonds was transposed on XB interactions involving C_6_At_6_ (and C_6_I_6_ for comparison purpose).

### 3.2. C_6_X_6_^…^NMe_3_ Complexes

In this section, we consider XB interactions between hexahalogenated benzene derivatives C_6_X_6_ (X = I or At) and trimethylamine (NMe_3_). NMe_3_ was selected since it is a strong Lewis base, widely used in studies dedicated to halogen bonding. The geometries of the X_5_C_6_–X^…^NMe_3_ systems (X = I, At) were optimized at the scalar relativistic PW6B95-D3/TZVPPD level of theory as well at the counterpoise-corrected (CP) MP2/TZVPPD level of theory. In order to evaluate the SOC effects (∆SO) on the XBs, 2c-PW6B95-D3 calculations were performed as well on the X_5_C_6_–X^…^NMe_3_ complexes (X = I, At). ∆SO was estimated as the difference between the 2c-PW6B95-D3/TZVPPD and sr-PW6B95-D3/TZVPPD results. The latter can be compared to the results obtained from single-point MP2 calculations, i.e., 2c-MP2/TZVPPD//2c-PW6B95-D3/TZVPPD and sr-MP2/TZVPPD//sr-PW6B95-D3/TZVPPD. The results are presented in Table 3.

All the studied systems exhibited a C_s_ symmetry. The C, X, and N atoms were almost aligned, i.e., the angle between the C, X, and N atoms was close to 180°. Moreover, the interaction distances, *d*_X_*…*_N_, were shorter than the sum of the van der Waals radii of N and X, with X = I, At (a radius of 2.02 Å, based on sr-calculations [70], is assumed for astatine). These geometric features were characteristic of XB interactions. The sr-PW6B95-D3/TZVPPD results were found to be in good agreement with the sr-MP2/TZVPPD ones. The interaction energies were slightly weaker, by ~3 kJ/mol, and consequently slightly longer interaction distances were predicted, by ~0.03 Å (1%). The agreement was even better if one considers the values of the C–X bond lengthening upon complexation or the values of the interaction angles. The XB mediated by astatine (At–XB) was stronger than the XB involving the iodine atom (I–XB), the difference of interaction energy being of ~8 kJ/mol. This trend was consistent with the assumption that an XB interaction strengthens with the increase in the polarizability and with the decrease in the electronegativity of the halogen element involved in the interaction.

The effects of the SOC were significantly different according to the halogen atom involved in the XB. Regarding I–XB, the interaction energy was negligibly affected (1%) as well as the interaction distance, according to the 2c-PW6B95-D3 calculations. This was not the case for At–XB. The consideration of the SOC yielded a weakening of the interaction energy by 4.9 kJ/mol, i.e., 12% (Table 3). The MP2 calculations consistently predicted a weakening of 8%. These results agreed with a lengthening of the At^…^N interaction distance that was ten times greater than in the case of the I–XB (0.069 Å vs. 0.007 Å). It is worth noting that the reduced ability of astatine to form XB interactions was expected from the previous discussion of the SOC effect on the nature of the C–At bond. Because the SOC enhances its CS character, the local electrophilicity was reduced at the astatine’s σ-hole according to the calculated 20% decrease in *ω*^+^_S,max_. In the same vein, it is worth noting that the *V*_S,max_ descriptor, commonly used to quantify the σ-hole strength [2,18], was reduced as well by ~6% when SOC was taken into account. Hence, both descriptors agreed with a decreased XB donating ability of astatine.

The weakening of the halogen bond donating ability of astatine upon inclusion of SOC led to a reduced energetic gap by comparison with the corresponding XB mediated by iodine. Thus, the At–XB was 3.9 kJ/mol stronger than the I–XB at the 2c-PW6B95-D3/TZVPPD level of theory, and the corresponding value was 4.6 kJ/mol at the 2c-MP2/TZVPPD//2c-PW6B95-D3/TZVPPD level of theory (Table 3). Hence, the hexaastatobenzene appears, at first, as a better XB donor than the hexaiodobenzene, even when the relativistic spin–orbit interaction is taken into account in quantum mechanical calculations. However, it has been shown in a previous study that, depending on the nature of the interacting Lewis base, the CAt_4_ species may or may not form stronger XBs than those formed by CI_4_ [32]. With the aim of verifying this behavior in the case of C_6_At_6_, we planned to study interactions with other Lewis bases. We realized that C_6_X_6_ species (X = I, At) were indeed Lewis bases through their π systems, acting as acceptor. In the next section, we extend the investigations by the consideration of XB complexes formed between two hexahalogenated benzenes.

### 3.3. C_6_X_6_^…^C_6_Y_6_ Complexes

**Figure 2:**
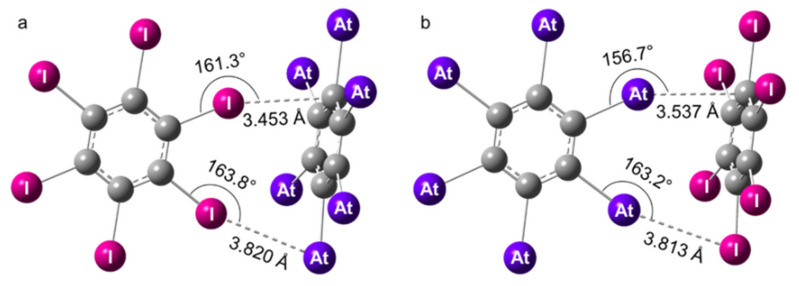
Structures of the XB complexes formed by C_6_At_6_ and C_6_I_6_ at the 2c-PW6B95-D3/TZVPPD level of theory. The XB interactions mediated by iodine (X = I, Y = At) are displayed on the left (**a**) and the ones mediated by astatine (X = At, Y = I) on the right (**b**).

In the XB complexes formed by two hexahalogenated benzenes (i.e., C_6_X_6_ and C_6_Y_6_ with X, Y = I, At), C_6_X_6_ represents the XB donor, whereas C_6_Y_6_ acts as the Lewis base (XB acceptor). Note that some similarities exist with the dimers of benzene, (C_6_H_6_)_2_, which have been widely studied as models of the interactions involving aromatic rings (π–π, π–hydrogen bond interactions) [71,72]. The accurate description of π–π interactions represents, as well, a challenge for the development of cost-effective quantum chemistry methods [73,74]. In our investigation of the C_6_X_6_^…^C_6_Y_6_ heterodimers, we left aside the question of π–π interactions (so-called sandwich and parallel-displaced structures) to focus on the structures stabilized by XB interactions. We will consider and compare the interactions mediated by iodine (X = I, Y = At) and by astatine (X = At, Y = I). The structure common to both types of interaction adopts a T-shaped tilted (TT) conformation with C_s_ symmetry, similar to the one described for the C_6_H_6_ dimers. No stable T-shaped (T) conformation could be evidenced. Note that the TT structure competes to be the most stable conformation in (C_6_H_6_)_2_ [74], and the stabilization of the TT conformation was here achieved through two interactions (see Figure 2). The first one (X^…^Y) occurred between one halogen atom of the XB donor (C_6_X_6_ with X = I, At) and the negative belt of a halogen atom in C_6_Y_6_ (Y = At, I). The second interaction (X^…^C) arose between another X atom and a part of the π system of C_6_Y_6_ located at the vertical of a carbon atom, owing to the structural constraints induced by the X^…^Y interaction and the rigidity of the C_6_X_6_ monomer.

At the scalar-relativistic level of theory, the PW6B95-D3/TZVPPD optimized geometries were found in good agreement with the ones obtained from counterpoise-corrected MP2/TZVPPD calculations (cf. Table S2 in Supplementary Materials). The distances corresponding to the X^…^Y interactions (*d*_X_*…*_Y_) were slightly shorter, on average by 2%, whereas the distances corresponding to the X^…^C interactions (*d*_X_*…*_C_) appeared a bit longer, by 4%. The structures of the dimers computed at the 2c-PW6B95-D3/TZVPPD level of theory are presented in Figure 2. The *d*_X_*…*_Y_ and *d*_X_*…*_C_ distances were, at least, 4% shorter than the sum of the van der Waals radii of the atoms involved in these interactions. The displayed interaction angles, of approximately 160°, significantly deviated from the optimum value of 180°, certainly because of the structural constraints mentioned above (accommodating two interactions plus the rigidity of the monomers). These C_6_X_6_^…^C_6_Y_6_ dimers were without a doubt XB complexes stabilized via two interactions of similar strengths. For instance, the *d*_At_*…*_C_ and the *d*_At_*…*_I_ distances were approximately 5% shorter than the sum of the van der Waals radii in the complex ruled by At–XBs (X = At, Y = I). In the case of X = I and Y = At, these were the interaction angles that were very similar (2.5° difference).

The most unusual and surprising result was that C_6_At_6_^…^C_6_I_6_ was not more stable than C_6_I_6_^…^C_6_At_6_, i.e., the XBs involving astatine were not stronger than their counterparts mediated by iodine. Indeed, the stabilization energy resulting from the two At–XBs in C_6_At_6_^…^C_6_I_6_ was −48.0 kJ/mol at the 2c-PW6B95D3/TZVPPD level of theory, which is nearly equal to the total interaction energy due to the I–XBs in C_6_I_6_^…^C_6_At_6_, −47.6 kJ/mol. The situation was even more unfavorable for At–XBs according to the 2c-MP2/TZVPPD calculations, the total interaction energy being −52.7 kJ/mol, while the one for I–XBs was −56.0 kJ/mol (3.3 kJ/mol stronger). Even though the deviations for the computed interaction energies between the two theory levels (i.e., 2c-PW6B95D3/TZVPPD and 2c-MP2/TZVPPD) can represent 8 kJ/mol, both methodologies agree on the tendency that XBs mediated by astatine may not be stronger than their iodine counterparts. This result is at variance with the general consensus that the most polarizable and less electronegative halogen elements form the strongest XBs [2,18]. As seen above, the C–At bonds in C_6_At_6_ exhibited a CS character that was enhanced by the relativistic spin–orbit interaction. Since the CS bonding weakened the local electrophilicity at the σ-hole (cf. Figure S1 in Supplementary Materials), the fact that At–XBs can become similar or weaker than their iodinated counterparts when the SOC is considered in the calculations reveals a connection to the CS character of the C–At bonds. The CS bonding did not only diminish the XB donating ability of C_6_At_6_, but it possibly changed it into a weaker XB donor than C_6_I_6_.

A fundamental question arising from the above results is whether the observed behavior is specific to C_6_At_6_ or if it can occur with other halogenated aromatic compounds. In this framework, the case of borirene, C_2_BH_3_, is interesting to consider for several reasons. It is the smallest representative of neutral aromatic molecules, consisting of a three-membered heterocyclic ring [75]. Additionally, the perhalogenation with astatine leads to the formation of both C–At and B–At bonds, two types of bond on which are currently based the most advanced radiosynthetic protocols for the use of ^211^At in targeted alpha therapy [76,77,78].

### 3.4. C_2_BX_3_^…^C_2_BY_3_ Complexes

**Figure 3:**
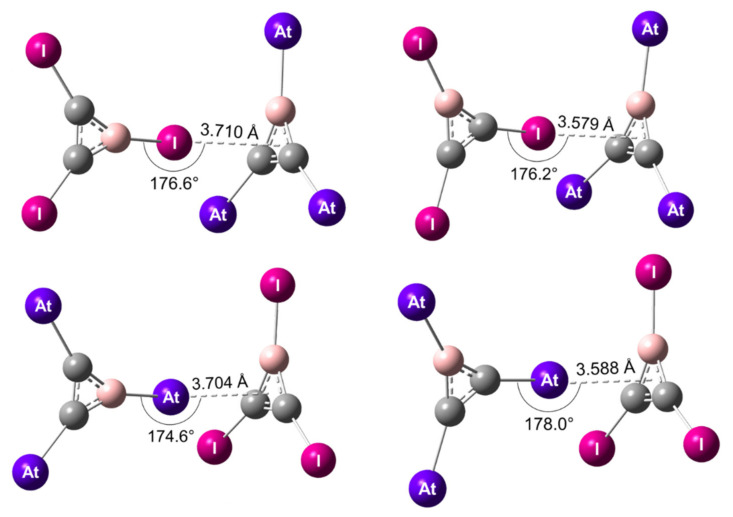
Structures of the XB complexes formed by C_2_BI_3_ and C_2_BAt_3_ at the 2c-PW6B95D3/TZVPPD level of theory. The XB interactions mediated by iodine (X = I, Y = At) are displayed on top and the ones mediated by astatine (X = At, Y = I) on the bottom.

In the C_2_BX_3_^…^C_2_BY_3_ heterodimers, the stabilization can occur through XB interactions mediated by astatine (X = At, Y = I) or iodine (X = I, Y = At). The X atom of the XB donor is pointing towards the centroid of the aromatic π system of the C_2_BY_3_ monomer. Since X can be bound to a carbon atom or to the boron atom, four types of XBs were studied and the corresponding most stable structures are presented in Figure 3.

Note that the DFT geometries optimized at the scalar-relativistic level agree well with those obtained from counterpoise-corrected sr-MP2/TZVPPD calculations. The interaction distances were slightly longer, by 3% on average, and therefore the interaction energies were slightly weaker, by ~1 kJ/mol (cf. Table S3 in Supplementary Materials). According to the 2c-PW6B95-D3/TZVPPD results, all the XB complexes exhibited a C_s_ symmetry. The angle between the R–X bond (R = B, C) and the centroid of the C_2_BY_3_ cycle was close to 180°, i.e., R, X, and the centroid were almost aligned (Figure 3). It is noteworthy to mention that the XB mediated by an iodine atom bound to the boron atom (I_B_–XB) showed a longer interaction distance, 3.710 Å, than that of the XB mediated by iodine but bound to a carbon atom (I_C–_XB), 3.579 Å. The same trend was noticed with the At–XBs. When the astatine atom was bound to carbon atoms, the At_C–_XB was 0.116 Å shorter than the corresponding At_B_–XB, i.e., when astatine was bound to the boron atom. It supports stronger I_C_–XB and At_C–_XB interactions with respect to their I_B_–XB and At_B_–XB counterparts, respectively.

The interaction energies of XB complexes at the 2c-PW6B95D3/TZVPPD and 2c-MP2/TZVPD levels of theory are presented in Table 4. These energies are in line with the discussion based on the structural parameters: the interaction energy for the I_C–_XB is computed approximately 2 kJ/mol stronger than for the I_B_–XB. A similar difference was found between At_C_–XB and At_B_–XB. This finding corroborates the general consensus that the more electron-withdrawing the R group bound to the X halogen atom (χ(B) < χ(C)), the stronger the interaction with nucleophilic sites [10,79]. The R inductive effect manifests itself in particular at the halogen σ-hole in C_2_BX_3_ (Figure S2 in Supplementary Materials). *V*_S,max_ was larger when I was bound to C atoms (113.7 kJ/mol) rather than to the B atom (72.4 kJ/mol), as *V*_S,max_ was larger when At was bound to C atoms (134.7 kJ/mol) rather than to the B atom (91.2 kJ/mol). The strongest interactions were therefore mediated by the halogens bound to C atoms.

The next question was whether astatine in C_2_BAt_3_ or iodine in C_2_BI_3_ leads to the strongest XB. According to the 2c-PW6B95-D3/TZVPPD calculations, the interaction energy for At_C–_XB was −15.6 kJ/mol, while the one corresponding to I_C–_XB was −15.8 kJ/mol, i.e., slightly better for iodine. The dominance of C_2_BI_3_ over C_2_BAt_3_ was slightly more pronounced at the 2c-MP2/TZVPPD level of theory, the I_C_–XB interaction being 0.8 kJ/mol stronger than its astatinated counterpart (Table 4). Note that these differences of interaction energies were less than 1 kJ/mol, which is lower than the usual accuracy of the methods used. However, both our DFT and ab initio calculations agreed on the same trend, which confirmed the previous results obtained on XB complexes between C_6_I_6_ and C_6_At_6_; the heaviest halogen element may not form stronger XB interactions than its lighter homologue. It must be stressed that this behavior cannot be anticipated from the value of the reactivity descriptors computed for C_2_BAt_3_ and C_2_BI_3_. Indeed, neither the comparison between computed *V*_S,max_ at the astatine and iodine σ-holes (Figure S2 in the Supplementary Materials) nor the comparison between computed *ω*^+^_S,max_ at the astatine and iodine σ-holes (Figure S3 in the Supplementary Materials) allow one to predict if At–XB interactions will be stronger or weaker than the corresponding I–XBs. Here again, the “unexpected” behavior was observed provided that the spin–orbit interaction was taken into account in the relativistic calculations. Indeed, the relative stability between the At_C–_XB and I_C–_XB interactions was inverted by ~0.9 kJ/mol, with respect to the sr results (Table S2 in the Supplementary Materials). Since SOC enhances the astatine propensity to form CS bonds [52,59] and C–At bonds can exhibit a significant CS character [32,52], it is unsurprising to notice in C_2_BAt_3_ a weakening of the local electrophilicity at the astatine’s σ-hole. The *ω*^+^_S,max_ value was reduced by 7% upon SOC (Figure S3 in the Supplementary Materials) and, consequently, the potential of astatine to accept charge transfers from XB acceptors.

In addition, the comparison between the interactions where the X halogen (X = I, At) was bound to the boron atom also revealed a similar behavior. First, the *ω*^+^_S,max_ value at the σ-hole of the astatine atom bound to B decreased by 4% upon SOC (Figure S3 in the Supplementary Materials). The 2c-PW6B95D3/TZVPPD or 2c-MP2/TZVPPD calculations therefore led to analogous stabilities for I_B_–XB and At_B_–XB interactions, the energy difference being less than 0.2 kJ/mol for a given level of theory (Table 4). Given the expected precision of the methods used, in these systems neither iodine nor astatine can be identified with certainty as the halogen mediating the strongest interaction.

## 4. Conclusions

In this work, the ability of C_6_I_6_ and C_6_At_6_ halobenzenes to form halogen-bond (XB) interactions was investigated by means of two-component relativistic quantum mechanical calculations. Although astatine is the less electronegative and the most polarizable of the halogen elements, it was shown that the strength of halogen bonds mediated by astatine (At–XBs) can be similar or weaker than one of their iodinated counterparts (I–XBs) when comparing complexes formed by C_6_At_6_ and C_6_I_6_. This behavior cannot be anticipated from the comparison between reactivity descriptors such as *V*_S,max_ computed at the astatine and iodine σ-holes of each XB donor. Conversely, some rationale is actually gained thanks to QTAIM and ELF topological analyses of the electronic structure of C_6_At_6_. Astatine being the heaviest halogen element, in the computations, we used the relativistic effects as a tool to disclose the bonding patterns in the C_6_At_6_ species. According to previous results by Alvarez-Thon and co-workers [26], the topological descriptors testify to a strong electron delocalization. But above all, the C–At bonds demonstrate a significant charge-shift (CS) character. CS bonding is a mechanism that aims to minimize Pauli repulsion between the bonding electrons and the σ lone-pair of astatine (referred to as the lone-pair bond-weakening effect, LPBWE [63]). The spin–orbit coupling (SOC) is known to enhance the astatine propensity for CS bonding [25,52,59], and the CS component of C–At bonds in C_6_At_6_ is indeed increased upon inclusion of SOC in relativistic calculations. If σ electrons suffer a stronger Pauli repulsion (LPBWE), the local electrophilicity at the astatine σ-hole is also significantly decreased, by ~20% according to the *ω*^+^_S,max_ value. This electrophilicity index is intended to probe the charge transfer contribution to the XB stabilization [31] and, indeed, the interactions were weakened with studied Lewis bases, according to two-component relativistic quantum mechanical calculations.

Signs of this connection between XB and CS bonding have been previously found in XB complexes involving either At_2_ or CAt_4_ [24,32]. The significance of the CS character of bonds in these XB donors translates into a weakened electrophilicity at the astatine σ-hole, and eventually weaker interactions than those involving their iodinated counterparts, I_2_ and CI_4_. In ancient Roman myth, Janus is the god of beginnings and endings. Just as Janus presents two faces, astatine seems to have two antinomic characters. On the basis of the features given above (i.e., electronegativity, polarizability), astatine in R–At compounds should be the strongest XB donor atom. However, its ability for CS bonding, if operating within the R–At bond, then hinders the formation of XBs. An obvious manifestation of this ambivalence is to find weaker At–XBs than their I–XB analogues. Currently, a handful of examples have been characterized. But the possibility of widespread interplay between halogen bonding and charge-shift bonding remains to be demonstrated, both concerning astatine and other halogens such as fluorine.

## Figures and Tables

**Table 1:** ELF electron population analysis of C_6_X_6_ species (X = I, At) obtained at the 2c-PW6B95-D3/TZVPPD level of theory.

	V(C, C): N	V(C, C): λ	C(X) + V(X): N	V(X): σ^2^	V(C, C): N	V(C, X): λ
X = I	2.90	0.48	24.30	1.03	1.72	0.61
∆SO ^a^	+0.03	−0.00	−0.22	−0.00	−0.08	+0.00
X = At	2.87	0.48	24.34	1.12	1.70	0.63
∆SO ^a^	+0.00	+0.00	+0.01	+0.00	−0.01	+0.01

^a^ Defined as the difference between the results of 2c-PW6B95-D3/TZVPPD and sr-PW6B95-D3/TZVPPD calculations.

**Table 2:** Selected QTAIM descriptors (in a.u.) obtained at the 2c-PW6B95-D3/TZVPPD level of theory for C–X bonds in C_6_X_6_ species (X = I, At).

	*ρ*_b_^b^	∇^2^*ρ*_b_^c^	|V_b_|/G_b_^d^	δ(C, X) ^e^	q(X) ^f^
X = I	0.13	−0.03	2.13	1.11	0.19
∆SO ^a^	−0.00	+0.00	−0.01	−0.01	−0.00
X = At	0.10	0.04	1.82	0.99	0.19
∆SO ^a^	−0.01	+0.01	−0.07	−0.09	−0.00

^a^ Defined as the difference between the results of 2c-PW6B95-D3/TZVPPD and sr-PW6B95-D3/TZVPPD calculations. ^b^ Electron density at the BCP. ^c^ Laplacian of the electron density at the BCP. ^d^ Ratio between the potential energy density and the (positive definite) kinetic energy density at the BCP. ^e^ Delocalization index. ^f^ Atomic charge.

**Table 3:** PW6B95-D3/TZVPPD and MP2/TZVPPD counterpoise corrected interaction energies and related parameters: interaction distances, lengthening of C–X bonds upon complexation, and interaction angles, for the X_5_C_6_–X^…^NMe_3_ complexes (X = I, At).

	Δ*E*^CP^ (kJ/mol)	*d*_X_*…*_N_ (Å)	∆*d*_C–X_ (Å)	α_CXN_ (°)
I–XB	−32.6	2.835	0.026	179.8
sr-PW6B95-D3				
At–XB	−40.9	2.826	0.036	179.7
I–XB	−32.6	2.799 ^a^	0.028 ^a^	179.7 ^a^
sr-MP2				
At–XB	−32.6	2.800 ^a^	0.037 ^a^	179.8 ^a^
I–XB	−32.1	2.842	0.024	180.0
∆SO	0.5	0.007	−0.002	0.2
2c-PW6B95-D3				
At–XB	−36.0	2.895	0.019	179.5
∆SO	4.9	0.069	−0.017	−0.2
I–XB	−35.3	–	–	–
∆SO ^b^	0.3	–	–	–
2c-MP2		–	–	–
At–XB	−39.9	–	–	–
∆SO ^b^	3.6	–	–	–

^a^ Counterpoise-corrected (CP) values. ^b^ Defined as the difference between the results of 2c-MP2/TZVPPD//2c-PW6B95-D3/TZVPPD and sr-MP2/TZVPPD//sr-PW6B95-D3/TZVPPD calculations.

**Table 4:** Counterpoise-corrected 2c-PW6B95D3/TZVPPD and 2c-MP2/TZVPPD interaction energies (ΔE^CP^) of the C_2_BX_3_^…^C_2_BY_3_ heterodimers with X, Y = I, At.

Δ*E*^CP^ (kJ/mol)	I_B_–XB	I_C–_XB	At_B_–XB	At_C–_XB
2c-PW6B95-D3/TZVPPD	−13.7	−15.8	−13.9	−15.6
2c-MP2/TZVPPD	−14.2 ^a^	−16.7^a^	−14.0 ^a^	−15.9 ^a^

^a^ 2c-MP2/TZVPPD//2c-PW6B95-D3/TZVPPD calculations.

## Data Availability

The data that support the findings of this study are available. Additional information can be requested from the corresponding author upon reasonable request.

## Supplementary Materials

The following are available online. Figure S1: 2c-PW6B95D3/TZVPPD calculated electrostatic potential and local electrophilicity at the C_6_At_6_ and C_6_I_6_ molecular surfaces; Figure S2: 2c-PW6B95D3/TZVPPD calculated electrostatic potential at the C_2_BI_3_ and C_2_BAt_3_ molecular surfaces; Figure S3: Local electrophilicity calculated at the 2c-PW6B95D3/TZVPPD level of theory at the C_2_BI_3_ and C_2_BAt_3_ molecular surfaces; Table S1: Selected QTAIM descriptors obtained at the 2c-PW6B95-D3/TZVPPD level of theory for C–C bonds in C_6_X_6_ species (X = I, At); Table S2: Interaction distances, lengthening of C–X bonds upon complexation, interaction angles, and directionality angles for the C_6_X_6_^…^C_6_Y_6_ complexes (X, Y = I, At) at the counterpoise-corrected sr-MP2/TZVPPD level of theory; Table S3: Scalar relativistic PW6B95-D3/TZVPPD and MP2/TZVPPD counterpoise corrected interaction energies and related parameters: interaction distances, lengthening of R–X bonds upon complexation (R = B, C), and interaction angles, for the C_2_BX_3_^…^C_2_BY_3_ complexes (X, Y = I, At).

## References

[1] Politzer P., Murray J.S., Clark T. (2013). Halogen Bonding and Other σ-Hole Interactions: A Perspective. Phys. Chem. Chem. Phys..

[2] Kolář M.H., Hobza P. (2016). Computer Modeling of Halogen Bonds and Other σ-Hole Interactions. Chem. Rev..

[3] Parisini E., Metrangolo P., Pilati T., Resnati G., Terraneo G. (2011). Halogen Bonding in Halocarbon–Protein Complexes: A Structural Survey. Chem. Soc. Rev..

[4] Lu Y., Liu Y., Xu Z., Li H., Liu H., Zhu W. (2012). Halogen Bonding for Rational Drug Design and New Drug Discovery. Expert Opin. Drug Discov..

[5] Wilcken R., Zimmermann M.O., Lange A., Joerger A.C., Boeckler F.M. (2013). Principles and Applications of Halogen Bonding in Medicinal Chemistry and Chemical Biology. J. Med. Chem..

[6] Mukherjee A., Tothadi S., Desiraju G.R. (2014). Halogen Bonds in Crystal Engineering: Like Hydrogen Bonds yet Different. Acc. Chem. Res..

[7] Berger G., Soubhye J., Meyer F. (2015). Halogen Bonding in Polymer Science: From Crystal Engineering to Functional Supramolecular Polymers and Materials. Polym. Chem..

[8] Bulfield D., Huber S.M. (2016). Halogen Bonding in Organic Synthesis and Organocatalysis. Chem. Eur. J..

[9] Li B., Zang S.-Q., Wang L.-Y., Mak T.C.W. (2016). Halogen Bonding: A Powerful, Emerging Tool for Constructing High-Dimensional Metal-Containing Supramolecular Networks. Coord. Chem. Rev..

[10] Cavallo G., Metrangolo P., Milani R., Pilati T., Priimagi A., Resnati G., Terraneo G. (2016). The Halogen Bond. Chem. Rev..

[11] Riley K.E., Merz K.M. (2007). Insights into the Strength and Origin of Halogen Bonding: The Halobenzene-Formaldehyde Dimer. J. Phys. Chem. A.

[12] Lu Y.-X., Zou J.-W., Wang Y.-H., Jiang Y.-J., Yu Q.-S. (2007). Ab Initio Investigation of the Complexes between Bromobenzene and Several Electron Donors: Some Insights into the Magnitude and Nature of Halogen Bonding Interactions. J. Phys. Chem. A.

[13] Riley K.E., Murray J.S., Fanfrlík J., Řezáč J., Solá R.J., Concha M.C., Ramos F.M., Politzer P. (2011). Halogen Bond Tunability I: The Effects of Aromatic Fluorine Substitution on the Strengths of Halogen-Bonding Interactions Involving Chlorine, Bromine, and Iodine. J. Mol. Model..

[14] Adasme-Carreño F., Muñoz-Gutierrez C., Alzate-Morales J.H. (2016). Halogen Bonding in Drug-like Molecules: A Computational and Systematic Study of the Substituent Effect. RSC Adv..

[15] Tsuzuki S., Uchimaru T., Wakisaka A., Ono T. (2016). Magnitude and Directionality of Halogen Bond of Benzene with C_6_F_5_X, C_6_H_5_X, and CF_3_X (X = I, Br, Cl, and F). J. Phys. Chem. A.

[16] Chernysheva M.V., Bulatova M., Ding X., Haukka M. (2020). Influence of Substituents in the Aromatic Ring on the Strength of Halogen Bonding in Iodobenzene Derivatives. Cryst. Growth Des..

[17] Otte F., Kleinheider J., Hiller W., Wang R., Englert U., Strohmann C. (2021). Weak yet Decisive: Molecular Halogen Bond and Competing Weak Interactions of Iodobenzene and Quinuclidine. J. Am. Chem. Soc..

[18] Clark T., Hennemann M., Murray J.S., Politzer P. (2007). Halogen Bonding: The σ-Hole. J. Mol. Model..

[19] Alkorta I., Blanco F., Solimannejad M., Elguero J. (2008). Competition of Hydrogen Bonds and Halogen Bonds in Complexes of Hypohalous Acids with Nitrogenated Bases. J. Phys. Chem. A.

[20] Hill J.G., Hu X. (2013). Theoretical Insights into the Nature of Halogen Bonding in Prereactive Complexes. Chem. Eur. J..

[21] Wilbur D.S. (2013). Enigmatic Astatine. Nat. Chem..

[22] Guo N., Maurice R., Teze D., Graton J., Champion J., Montavon G., Galland N. (2018). Experimental and Computational Evidence of Halogen Bonds Involving Astatine. Nat. Chem..

[23] Liu L., Guo N., Champion J., Graton J., Montavon G., Galland N., Maurice R. (2020). Towards a Stronger Halogen Bond Involving Astatine: Unexpected Adduct with Bu_3_PO Stabilized by Hydrogen Bonding. Chem. Eur. J..

[24] Graton J., Rahali S., Questel J.-Y.L., Montavon G., Pilmé J., Galland N. (2018). Spin–Orbit Coupling as a Probe to Decipher Halogen Bonding. Phys. Chem. Chem. Phys..

[25] Sarr S., Graton J., Rahali S., Montavon G., Galland N. (2021). Delocalized Relativistic Effects, from the Viewpoint of Halogen Bonding. Phys. Chem. Chem. Phys..

[26] Ramírez-Tagle R., Alvarado-Soto L., Villavicencio-Wastavino A., Alvarez-Thon L. (2016). Relativistic Effects on the Aromaticity of the Halogenated Benzenes: C_6_X_6_, X = H, F, Cl, Br, I, At. Phys. Chem. Chem. Phys..

[27] Mitin A.V., van Wüllen C. (2006). Two-Component Relativistic Density-Functional Calculations of the Dimers of the Halogens from Bromine through Element 117 Using Effective Core Potential and All-Electron Methods. J. Chem. Phys..

[28] Yang D.-D., Wang F. (2012). Structures and Stabilities of Group 17 Fluorides EF_3_ (E = I, At, and Element 117) with Spin-Orbit Coupling. Phys. Chem. Chem. Phys..

[29] Frisch M.J., Trucks G.W., Schlegel H.B., Scuseria G.E., Robb M.A., Cheeseman J.R., Scalmani G., Barone V., Petersson G.A., Nakatsuji H. (2016). Gaussian 16 Rev. A.03.

[30] Sergentu D.-C., David G., Montavon G., Maurice R., Galland N. (2016). Scrutinizing “Invisible” Astatine: A Challenge for Modern Density Functionals. J. Comput. Chem..

[31] Galland N., Montavon G., Questel J.-Y.L., Graton J. (2018). Quantum Calculations of At-Mediated Halogen Bonds: On the Influence of Relativistic Effects. New J. Chem..

[32] Sarr S., Graton J., Montavon G., Pilmé J., Galland N. (2020). On the Interplay between Charge-Shift Bonding and Halogen Bonding. ChemPhysChem.

[33] Grimme S., Ehrlich S., Goerigk L. (2011). Effect of the Damping Function in Dispersion Corrected Density Functional Theory. J. Comput. Chem..

[34] Peterson K.A. (2003). Systematically Convergent Basis Sets with Relativistic Pseudopotentials. I. Correlation Consistent Basis Sets for the Post-d Group 13–15 Elements. J. Chem. Phys..

[35] Peterson K.A., Shepler B.C., Figgen D., Stoll H. (2006). On the Spectroscopic and Thermochemical Properties of ClO, BrO, IO, and Their Anions. J. Phys. Chem. A.

[36] Weigend F., Baldes A. (2010). Segmented contracted basis sets for one- and two-component Dirac–Fock effective core potentials. J. Chem. Phys..

[37] Weigend F., Ahlrichs R. (2005). Balanced basis sets of split valence, triple zeta valence and quadruple zeta valence quality for H to Rn: Design and assessment of accuracy. Phys. Chem. Chem. Phys..

[38] Rappoport D., Furche F. (2010). Property-optimized Gaussian basis sets for molecular response calculations. J. Chem. Phys..

[39] Boys S., Bernardi F. (1970). The calculation of small molecular interactions by the differences of separate total energies. Some procedures with reduced errors. Mol. Phys..

[40] TURBOMOLE A Development of University of Karlsruhe and Forschungszentrum Karlsruhe GmbH, 1989–2007, TURBOMOLE GmbH, Since 2007. http://www.turbomole.com.

[41] Chattaraj P.K., Sarkar A.U., Roy D.R. (2006). Electrophilicity Index. Chem. Rev..

[42] Becke A.D., Edgecombe E.K. (1990). A simple measure of electron localization in atomic and molecular systems. J. Chem. Phys..

[43] Silvi B., Savin A. (1994). Classification of chemical bonds based on topological analysis of electron localization functions. Nat. Cell Biol..

[44] Gillespie R.J., Robinson E.A. (2006). Gilbert N. Lewis and the chemical bond: The electron pair and the octet rule from 1916 to the present day. J. Comput. Chem..

[45] Llusar R., Beltrán A., Andrés J., Noury S., Silvi B. (1999). Topological analysis of electron density in depleted homopolar chemical bonds. J. Comput. Chem..

[46] Bader R.F.W. (1994). Atoms in Molecules: A Quantum Theory.

[47] Koritsanszky T.S., Coppens P. (2001). Chemical Applications of X-ray Charge-Density Analysis. Chem. Rev..

[48] Matta C.F., Boyd R.J. (2007). The Quantum Theory of Atoms in Molecules: From Solid State to DNA and Drug Design.

[49] Bader R.F.W., Essen H. (1984). The characterization of atomic interactions. J. Chem. Phys..

[50] Matta C.F., Boyd R.J. (2007). An Introduction to the Quantum Theory of Atoms in Molecules. The Quantum Theory of Atoms in Molecules.

[51] Fradera X., Poater J., Simon S., Duran M., Solà M. (2002). Electron-pairing analysis from localization and delocalization indices in the framework of the atoms-in-molecules theory. Theor. Chem. Accounts.

[52] Pilmé J., Renault E., Bassal F., Amaouch M., Montavon G., Galland N. (2014). QTAIM Analysis in the Context of Quasirelativistic Quantum Calculations. J. Chem. Theory Comput..

[53] Pilmé J., Renault E., Ayed T., Montavon G., Galland N. (2012). Introducing the ELF Topological Analysis in the Field of Quasirelativistic Quantum Calculations. J. Chem. Theory Comput..

[54] Kozlowski D., Pilmé J. (2011). New insights in quantum chemical topology studies using numerical grid-based analyses. J. Comput. Chem..

[55] Amaouch M., Montavon G., Galland N., Pilmé J. (2015). What can tell the quantum chemical topology on carbon—Astatine bonds?. Mol. Phys..

[56] Amaouch M., Renault E., Montavon G., Galland N., Pilmé J., Chauvin R., Lepetit C., Silvi B., Alikhani E. (2016). Quantum Chemical Topology in the Field of Qua-sirelativistic Quantum Calculations. Applications of Topological Methods in Molecular Chemistry.

[57] Ayed T., Pilmé J., Teze D., Bassal F., Barbet J., Chérel M., Champion J., Maurice R., Montavon G., Galland N. (2016). 211 At-labeled agents for alpha-immunotherapy: On the in vivo stability of astatine-agent bonds. Eur. J. Med. Chem..

[58] Amaouch M., Sergentu D.-C., Steinmetz D., Maurice R., Galland N., Pilmé J. (2017). The bonding picture in hypervalent XF3(X = Cl, Br, I, At) fluorides revisited with quantum chemical topology. J. Comput. Chem..

[59] Pech C.G., Haase P.A.B., Sergentu D., Borschevsky A., Pilmé J., Galland N., Maurice R. (2020). Quantum chemical topology at the spin–orbit configuration interaction level: Application to astatine compounds. J. Comput. Chem..

[60] Noury S., Colonna F., Savin A., Silvi B. (1998). Analysis of the delocalization in the topological theory of chemical bond. J. Mol. Struct..

[61] Savin A., Silvi B., Colonna F. (1996). Topological analysis of the electron localization function applied to delocalized bonds. Can. J. Chem..

[62] Shaik S., Maitre P., Sini G., Hiberty P.C. (1992). The charge-shift bonding concept. Electron-pair bonds with very large ionic-covalent resonance energies. J. Am. Chem. Soc..

[63] Shaik S., Danovich D., Wu W., Hiberty P.C. (2009). Charge-shift bonding and its manifestations in chemistry. Nat. Chem..

[64] Shaik S., Danovich D., Silvi B., Lauvergnat D.L., Hiberty P.C. (2005). Charge-Shift Bonding—A Class of Electron-Pair Bonds That Emerges from Valence Bond Theory and Is Supported by the Electron Localization Function Approach. Chem. Eur. J..

[65] Outeiral C., Vincent M.A., Pendás M., Popelier P.L.A. (2018). Revitalizing the concept of bond order through delocalization measures in real space. Chem. Sci..

[66] Silvi B., Gillespie R.J., Gatti C., Reedijk J., Poeppelmeier K. (2013). Electron Density Analysis. Comprehensive Inorganic Chemistry II.

[67] Zhang L., Ying F., Wu W., Hiberty P.C., Shaik S. (2009). Topology of Electron Charge Density for Chemical Bonds from Valence Bond Theory: A Probe of Bonding Types. Chem. Eur. J..

[68] Réal F., Gomes A.S.P., Martínez Y.O.G., Ayed T., Galland N., Masella M., Vallet V. (2016). Structural, dynamical, and transport properties of the hydrated halides: How do At− bulk properties compare with those of the other halides, from F− to I−?. J. Chem. Phys..

[69] Cremer D., Kraka E. (1984). Chemical Bonds without Bonding Electron Density ? Does the Difference Electron-Density Analysis Suffice for a Description of the Chemical Bond?. Angew. Chem. Int. Ed..

[70] Mantina M., Chamberlin A.C., Valero R., Cramer C., Truhlar D.G. (2009). Consistent van der Waals Radii for the Whole Main Group. J. Phys. Chem. A.

[71] Meyer E.A., Castellano R.K., Diederich F. (2003). Interactions with Aromatic Rings in Chemical and Biological Recognition. Angew. Chem. Int. Ed..

[72] Hoeben F.J.M., Jonkheijm P., Meijer E.W., Schenning A.P.H.J. (2005). About Supramolecular Assemblies of π-Conjugated Systems. Chem. Rev..

[73] Černý J., Hobza P. (2007). Non-covalent interactions in biomacromolecules. Phys. Chem. Chem. Phys..

[74] Pitoňák M., Neogrády P., Řezáč J., Jurečka P., Urban M., Hobza P. (2008). Benzene Dimer: High-Level Wave Function and Density Functional Theory Calculations. J. Chem. Theory Comput..

[75] Balucani N., Asvany O., Lee A.Y.T., Kaiser R.I., Galland N., Hannachi Y. (2000). Observation of Borirene from Crossed Beam Reaction of Boron Atoms with Ethylene. J. Am. Chem. Soc..

[76] Wilbur D. (2008). [211At]Astatine-Labeled Compound Stability: Issues with Released [211At]Astatide and Development of Labeling Reagents to Increase Stability. Curr. Radiopharm..

[77] Vaidyanathan G., Zalutsky M.R. (2011). Applications of 211At and 223Ra in Targeted Alpha-Particle Radiotherapy. Curr. Radiopharm..

[78] Meyer G.-J. (2018). Astatine. J. Label. Compd. Radiopharm..

[79] Politzer P., Murray J.S., Clark T. (2010). Halogen bonding: An electrostatically-driven highly directional noncovalent interaction. Phys. Chem. Chem. Phys..

