# Supplementary material for: Astatine Facing Janus: Halogen Bonding vs. Charge-Shift Bonding
Source: Molecules. 2021 Jul 28;26(15):4568. doi: 10.3390/molecules26154568 (PMC8347445; doi:10.3390/molecules26154568)
Supplement: Supplementary file 1 [file molecules-26-04568-s001.zip › molecules-1298386-supplementary.pdf]

# Supplementary Materials

## Astatine facing Janus: halogen bonding vs. charge-shift bonding

Serigne Sarr, Julien Pilmé, Gilles Montavon, Jean-Yves Le Questel and Nicolas Galland

## Index


Page 2 **Figure S1.** 2c-PW6B95D3/TZVPPD calculated electrostatic potential (in kJ.mol$^{-1}$) and local electrophilicity (in a.u) at the $C_6At_6$ and $C_6I_6$ molecular surfaces.

Page 3 **Figure S2.** 2c-PW6B95D3/TZVPPD calculated electrostatic potential at the $C_2BI_3$ and $C_2BAt_3$ molecular surfaces.

Page 4 **Figure S3.** Local electrophilicity (in u.a), at the $C_2BI_3$ and $C_2BAt_3$ molecular surfaces calculated at the 2c-PW6B95D3/TZVPPD level of theory.

Page 5 **Table S1.** Selected QTAIM descriptors (in a.u.) obtained at the 2c-PW6B95-D3/TZVPPD level of theory for C–C bonds in $C_6X_6$ species (X = At, I).

**Table S2.** Interaction distances, lengthening of C–X bonds upon complexation, interaction angles and directionality angles for the $C_6X_6 \cdots C_6Y_6$ complexes (X, Y = I, At) at the counterpoise-corrected sr-MP2/TZVPPD level of theory.

Page 6 **Table S3.** Scalar relativistic PW6B95-D3/TZVPPD and MP2/TZVPPD counterpoise corrected interaction energies and related parameters: interaction distances, lengthening of R–X bonds upon complexation (R = B, C), and interaction angles, for the $C_2BX_3 \cdots C_2BY_3$ complexes (X, Y = I, At).

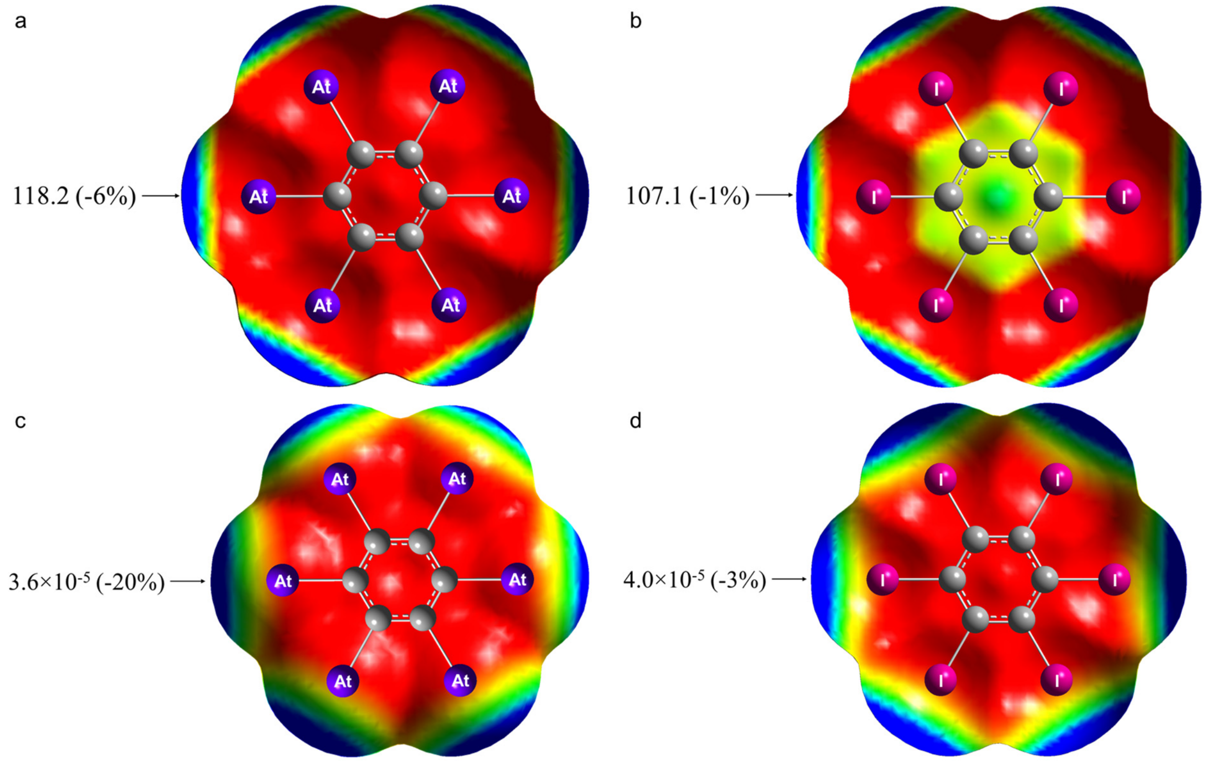


**Figure S1.** 2c-PW6B95D3/TZVPPD calculated electrostatic potential (a, b) and local electrophilicity (c, d) at the $C_6At_6$ and $C_6I_6$ molecular surfaces (defined by 0.001 a.u. density isosurfaces). The maximum values, $V_{S,max}$ and $\omega^{+}_{S,max}$, at the astatine and iodine σ-holes are in kJ.mol$^{-1}$ and a.u., respectively. Values in parenthesis represent the SOC effects. Color code: from red (lowest values) to blue (highest values).

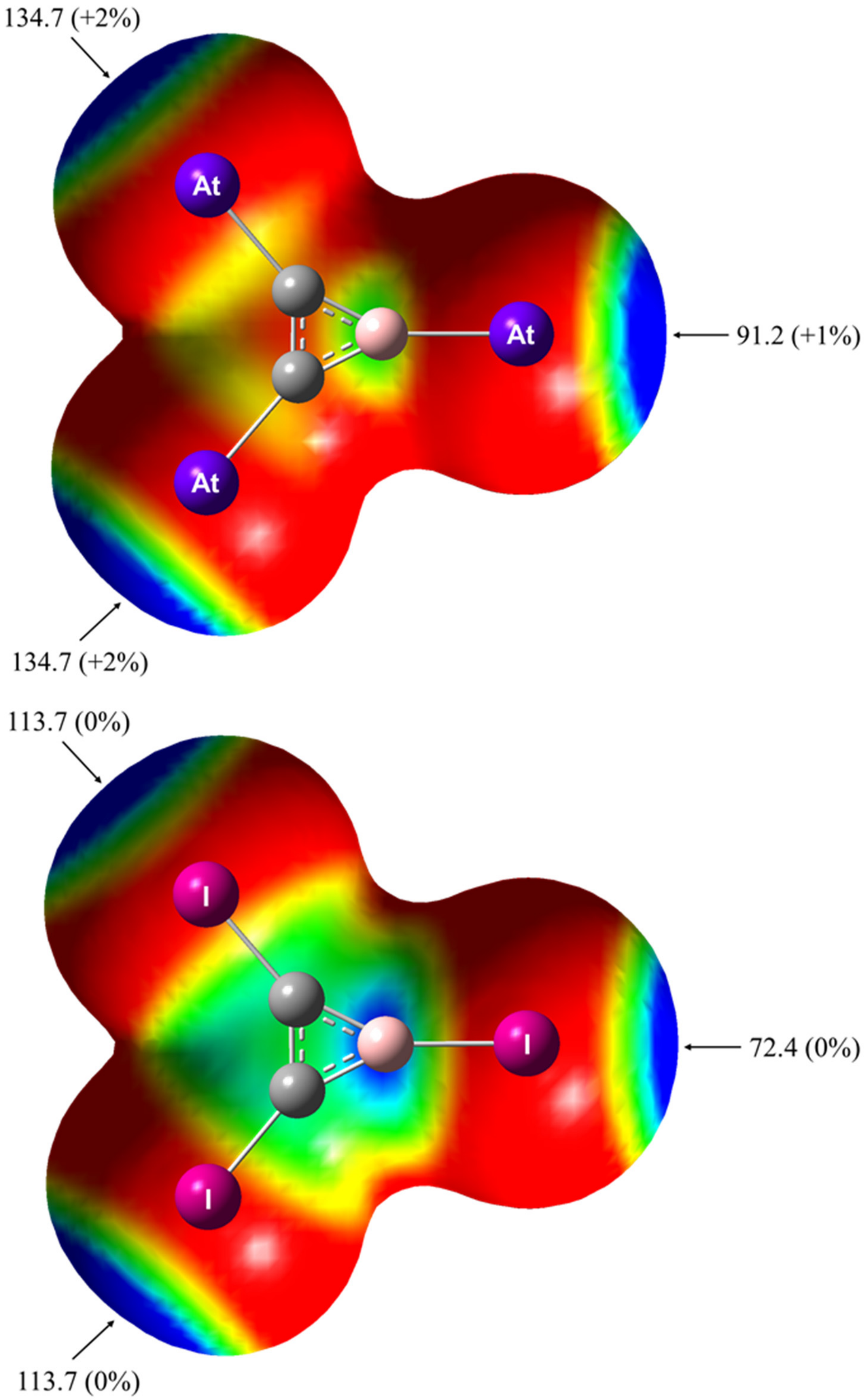


**Figure S2.** 2c-PW6B95D3/TZVPPD calculated electrostatic potential at the $C_2BI_3$ and $C_2BAt_3$ molecular surfaces (defined by 0.001 a.u. density isosurfaces). The maximum values, $V_{S,max}$, at the astatine and iodine σ-holes are in kJ.mol$^{-1}$ (SOC effects are given in parenthesis). Color code: from red (most negative values) to blue (most positive values).

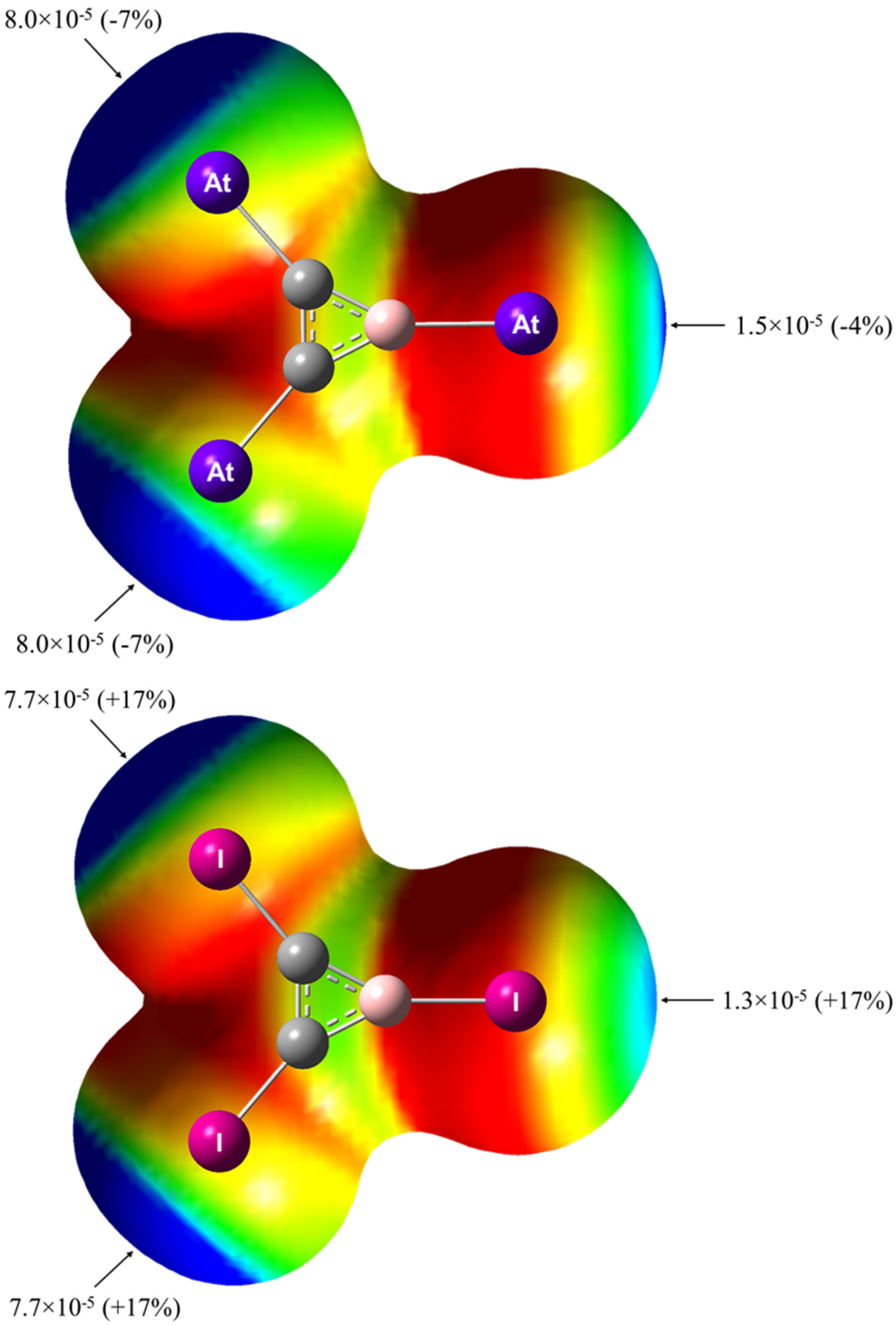


**Figure S3.** Local electrophilicity, at the $C_2BI_3$ and $C_2BAt_3$ molecular surfaces (defined by the 0.001 a.u. density isosurface) calculated at the 2c-PW6B95D3/TZVPPD level of theory. The most positive electrophilicity values, $\omega^{+}_{S,max}$, at the astatine and iodine σ-holes are in a.u. Values in parenthesis represent SOC effects are given in parenthesis. Color code: from red (lowest values) to blue (highest values).

**Table S1.** Selected QTAIM descriptors (in a.u.) obtained at the 2c-PW6B95-D3/TZVPPD level of theory for C–C bonds in $C_6X_6$ species (X = I, At).

| | $\rho_b$[b] | $\nabla^2\rho_b$[c] | $\lvert V_b\rvert/G_b$[d] | δ(C, C)[e] | FLU[f] |
|---|---|---|---|---|---|
| X = I | 0.31 | -0.90 | 4.05 | 1.35 | 0.0057 |
| ΔSO[a] | +0.00 | -0.02 | +0.02 | +0.00 | -0.0005 |
| | | | | | |
| X = At | 0.32 | -0.92 | 3.96 | 1.38 | 0.0004 |
| ΔSO[a] | +0.01 | -0.05 | -0.03 | +0.02 | -0.0033 |

[a] Defined as the difference between the results of 2c-PW6B95-D3/TZVPPD and sr-PW6B95-D3/TZVPPD calculations. [b] Electron density at the BCP. [c] Laplacian of the electron density at the BCP. [d] Ratio between the potential energy density and the (positive definite) kinetic energy density at the BCP. [e] Delocalization index. [f] Aromaticity index FLU= $\sum_1^n \left(\frac{\delta(C, C) - \delta_{ref}(C, C)}{\delta_{ref}(C, C)}\right)^2$ with *n* equal to the number of members in the ring, and $\delta_{ref}$(C,C) = 1.388 according to PW6B95-D3/TZVPPD calculations on the benzene molecule. Low values indicate a strong aromaticity character.

**Table S2.** Interaction distances, lengthening of C–X bonds upon complexation, interaction angles and directionality angles for the $C_6X_6\cdots C_6Y_6$ complexes (X, Y = I, At) at the counterpoise-corrected sr-MP2/TZVPPD level of theory.

| | **X = I, Y = At** | | **X = At, Y = I** | |
|---|---|---|---|---|
| | **I···C** | **I···At** | **At···C** | **At···I** |
| $d_{X\cdots C}$ (Å) | 3.373 | - | 3.400 | - |
| $d_{X\cdots Y}$ (Å) | - | 3.903 | - | 3.784 |
| $\Delta d_{C\text{–}X}$ (Å) | 0.005 | 0.005 | 0.007 | 0.007 |
| $\alpha_{CXC}$ (°) | 164.8 | 160.0 | 162.5 | 159.4 |
| $\alpha_{XYC}$ (°) | 109.4 | 68.2 | 103.4 | 72.7 |

**Table S3.** Scalar relativistic PW6B95-D3/TZVPPD and MP2/TZVPPD counterpoise corrected interaction energies and related parameters: interaction distances, lengthening of R–X bonds upon complexation (R = B, C), and interaction angles, for the $C_2BX_3 \cdots C_2BY_3$ complexes (X, Y = I, At).

| | | $\Delta E^{CP}$ (kJ/mol) | $d_{X\cdots N}$ (Å) | $\Delta d_{C-X}$ (Å) | $\alpha_{CXN}$ (°) |
|---|---|---|---|---|---|
| sr-PW6B95-D3 | $I_B$–XB | -13.3 | 3.711 | 0.000 | 172.3 |
| | $I_C$–XB | -14.9 | 3.597 | 0.000 | 178.1 |
| | $At_B$–XB | -13.8 | 3.712 | 0.001 | 173.1 |
| | $At_C$–XB | -15.4 | 3.587 | 0.001 | 175.5 |
| sr-MP2 | $I_B$–XB | -14.3 | 3.610[a] | 0.001[a] | 173.0[a] |
| | $I_C$–XB | -16.5 | 3.481[a] | 0.002[a] | 177.5[a] |
| | $At_B$–XB | -14.4 | 3.622[a] | 0.003[a] | 173.1[a] |
| | $At_C$–XB | -16.5 | 3.485[a] | 0.003[a] | 177.2[a] |

[a] Counterpoise-corrected values.
